# *Toxins* Best Paper Award 2015

**DOI:** 10.3390/toxins7030971

**Published:** 2015-03-19

**Authors:** Vernon L. Tesh

**Affiliations:** *Editor-in-Chief*, Department of Microbial and Molecular Pathogenesis, Medical Research and Education Building, Room 3002, College of Medicine, Texas A&M University System Health Science Center, 8447 State Highway 47, Bryan, TX 77807, USA; E-Mail: tesh@medicine.tamhsc.edu

In order to recognize outstanding papers related to biotoxins and toxinology that have been published in *Toxins*, the Editorial Board established an annual “*Toxins* Best Paper Award”. We are pleased to announce the first “*Toxins* Best Paper Award” for 2015. Nominations were selected by the Editorial Board members, with all papers published in 2011 eligible for consideration. Reviews and original research articles were evaluated separately. Following review and voting by the *Toxins* Best Paper Award Committee, the following three papers have won *Toxins* Best Paper Awards for 2015:

**Original Research Article Award**
  The following two research articles will share the award:
  Baozhu Guo, Natalie D. Fedorova, Xiaoping Chen, Chun-Hua Wan, Wei Wang, William C. Nierman, Deepak Bhatnagar and Jiujiang Yu *
    Gene Expression Profiling and Identification of Resistance Genes to *Aspergillus flavus* Infection in Peanut through EST and Microarray Strategies
    *Toxins* **2011**, *3*(7), 737-753; doi:10.3390/toxins3070737
    Available online: http://www.mdpi.com/2072-6651/3/7/737
  Mohamed Anwar Bin-Umer, John E. McLaughlin, Debaleena Basu, Susan McCormick and Nilgun E. Tumer *
    Trichothecene Mycotoxins Inhibit Mitochondrial Translation—Implication for the Mechanism of Toxicity
    *Toxins* **2011**, *3*(12), 1484-1501; doi:10.3390/toxins3121484
    Available online: http://www.mdpi.com/2072-6651/3/12/1484
**Review Award**
  Susan P. McCormick *, April M. Stanley, Nicholas A. Stover and Nancy J. Alexander
    Trichothecenes: From Simple to Complex Mycotoxins
    *Toxins* **2011**, *3*(7), 802-814; doi:10.3390/toxins3070802
    Available online: http://www.mdpi.com/2072-6651/3/7/802

Members of the *Toxins* Best Paper Award Committee described the article “Gene Expression Profiling and Identification of Resistance Genes to *Aspergillus flavus* Infection in Peanut through EST and Microarray Strategies” as “…this innovative study not only sheds light on the infection process, and associated resistance, but represents an excellent starting point for future development of tools to monitor aflatoxins, and improve crops with respect to this widespread fungal pathogen…”. The article “Trichothecene Mycotoxins Inhibit Mitochondrial Translation-Implication for the Mechanism of Toxicity” represents “…an important finding with respect to understanding these widespread toxins…. The study defines a novel mode of action, demonstrating that in addition to cytosolic translation, mitochondrial translation is a primary target of trichothecene mycotoxins.” The review “Trichothecenes: from Simple to Complex Mycotoxins” is “a concise, yet comprehensive, overview of trichothecene mycotoxin biosynthesis and classification”.

These three papers are valuable contributions to *Toxins*, as well as to the toxinology field in general. On behalf of the *Toxins* Best Paper Award Committee and the Editorial Board of *Toxins*, I congratulate the winners for their significant contributions and thank them for choosing to publish their best work in *Toxins*. In recognition of their accomplishments, both Dr. Jiujiang Yu and Dr. Nilgun E. Tumer will receive a prize of 500 CHF, as well as an opportunity to publish an additional paper free of charge in open access format in *Toxins*, after the standard peer-review process. Dr. Susan P. McCormick will be awarded the privilege of publishing an additional research paper, with all publication fees waived, in Open Access format in *Toxins*, after the standard peer-review procedure.

The Editorial Board and Editorial Staff at *Toxins* is committed to meeting the needs of the toxin research community by providing useful and timely reviews of all manuscripts submitted, and providing an open access journal for your results. Please consider submitting your work to *Toxins*, and I look forward to announcing your paper as a *Toxins* Best Paper in the future.

## *Toxins* Best Paper Award Committee

*Editor-in-Chief*
  Prof. Dr. Vernon L. Tesh
  Department of Microbial and Molecular Pathogenesis, Medical Research and Education Building, Room 3002, College of Medicine, Texas A&M University System Health Science Center, 8447 State Highway 47, Bryan, TX 77807, USA
  E-Mail: tesh@medicine.tamhsc.edu
  *Editor-in-Chief* of Section “Bacterial Toxins”
Founding *Editor-in-Chief*
  Prof. Dr. Med. Florian Lang
  Physiologisches Institut I, Universität Tübingen, Gmelinstrasse 5, D-72076 Tübingen, Germany
  E-Mail: florian.lang@uni-tuebingen.de
Section *Editor-in-Chief* of “Marine and Freshwater Toxins”
  Prof. Dr. John P. Berry
  Department of Chemistry and Biochemistry, Florida International University (FIU), 354/332 Marine Science, Biscayne Bay Campus, 3000 NE 151st St., North Miami, FL 33181, USA
  E-Mail: john.berry@fiu.edu
Prof. Massimo Reverberi
  Department of Environmental Biology, Sapienza University, Roma, P.le Aldo Moro 5, 00185 Roma, Italy
  E-Mail: massimo.reverberi@uniroma1.it

